
JOURNAL INFORMATION
==============================
NLM Title Abbreviation: ScientificWorldJournal
Journal ID: ScientificWorldJournal
NLM Journal ID: 101131163
Journal ID: TSWJ

The Scientific World Journal

ISSN: 2356-6140
EISSN: 1537-744X
Publisher: Wiley

ARTICLE INFORMATION
==============================
PMCID: PMC6084726
PMID: 30147468
DOI: 10.1100/tsw.2001.23.156
Article ID: 856414
Article version: 1
Subjects: Short Report

Recent Progress in the Identification of the X-Linked Infantile Spinal Muscular Atrophy (Xl-SMA) Gene: Implications for Neuronal Apoptosis

Baumbach L. L. 1 *
Dressman D. 2 3
Basterrechea H. 1
Ahearn M. E. 1
Bech-Hansen N. T. 4
Clark R. D. 5
Meind A. 6
Hoffman E. 2
Department of Pediatrics, University of Miami Sch Medicine, PO Box 016820, Miami, FL., 33136, USA
Children’s National Medical Center, Washington, D.C, USA
University of Pittsburgh, Pittsburgh, PA, USA
University of Calgary, Calgary, Alberta, Canada
USC Cancer Center, Los Angeles, CA, USA
Ludwig Maximilians Universitat, Munich, Germany
*L. L. Baumbach: lbaumbac@med.miami.edu
Publication date: 2001
Electronic publication date: 2001 Oct 12
Volume: 1
First page: 33
Last page: 33
Copyright: Copyright © 2001 L.L. Baumbach et al.
Copyright year: 2001
License: This is an open access article distributed under the Creative Commons Attribution License, which permits unrestricted use, distribution, and reproduction in any medium, provided the original work is properly cited.
License URL: https://creativecommons.org/licenses/by/3.0/

Funding: Muscular Dystrophy Association

==============================
Miami *Nature Biotechnology* Short Reports
*TheScientificWorld* (2001) 1(S3), 33SR
ISSN 1532-2246; DOI 10.110/tsw.2001.156

### RECENT PROGRESS IN THE IDENTIFICATION OF THE X-LINKED INFANTILE SPINAL MUSCULAR ATROPHY (*XL-SMA*) GENE: IMPLICATIONS FOR NEURONAL APOPTOSIS

L.L. Baumbach[1,]*, D. Dressman[2,3], H. Basterrechea[1], M.E. Ahearn[1], N.T. Bech-Hansen[4], R.D. Clark[5], A. Meindl[6], E. Hoffman[2]
[1]Dept of Pediatrics, Univ Miami Sch Medicine, PO Box 016820, Miami, FL., 33136; [2]Children's National Medical Center, Washington, D.C; [3]Univ. of Pittsburgh, Pittsburgh, PA; [4]University of Calgary, Calgary, Alberta; [5]USC Cancer Center, Los Angeles, CA; [6]Ludwig Maximilians Universtat, Munich, Germany
* lbaumbac@med.miami.edu

**INTRODUCTION**. Autosomal recessive spinal muscular atrophy (SMA) is characterized by progressive loss/atrophy of lower motor neurons. The most common presentations range from a lethal, infantile severe form associated with *in utero* anterior horn cell loss (i.e., Werdnig-Hoffman disease) to less severe, later-onset forms (i.e., Kugleberg-Welander disease), also associated with loss of anterior horn cells in the spinal cord [1]. The vast majority of autosomal recessive SMA cases map to a locus at 5q11.2, however, genetic heterogeneity has been noted. We have previously reported an X-linked recessive form of infantile lethal motor neuron disease (MIM 30021)[2], which closely resembles Werdnig-Hoffman disease, except for additional features of early onset or congenital contractures and/or fractures, in a single family that mapped to Xp11.3-Xq11.2. Here we report clinical and genetic data for eleven additional unrelated families identified in North American and Central and Western Europe, as well as our recent progress in disease gene identification.

**METHODS.** Families were recruited based on established diagnostic criteria, and five of the six new families evaluated for linkage to the candidate disease gene region, Xp11.3-q11.2. The remaining two families were not adequate for generation of a significant LOD score. Genomic DNA was analyzed using X-chromosome highly polymorphic markers. Concordance analysis was used to define maternal meiotic recombination breakpoints surrounding a disease gene region, followed by multipoint linkage analysis with additional DNA markers[2]. Results were interpreted using the *LINKAGE* and *VITESSE* programs.

**RESULTS.** Six families have been tested for linkage on the X-chromosome; all of these mapped to the same region as the first reported family[2]. One of the six families is thought to represent a new mutational event, and although was informative for the DNA markers used, and highly suggestive of positive linkage to the same region, was not included in LOD score calculations. **The cumulative LOD score for the five families is 7.3 at a = 0.0 in the Xp11.3-q11.2 region**. Further multipoint linkage analysis using these families has narrowed the candidate disease gene interval to a region defined by DXS8035-DXS1194. These results strongly support the existence of a major disease locus for XL-SMA between DXS 8026-DXS 7132 (Xp11.3-Xq11.3).

**DISCUSSION.** A complete transcript map fromXp11.3- centromere is being completed[3]. Indicated in the figure is the *XL- SMA* candidate region, with a diagram of DNA markers used for linkage analysis, and an approximate number of genes in sub-regions. We are now focusing our efforts on refined linkage analysis (to continue to narrow the candidate region), and candidate gene identification and mutation detection. Our latest findings will be reported.

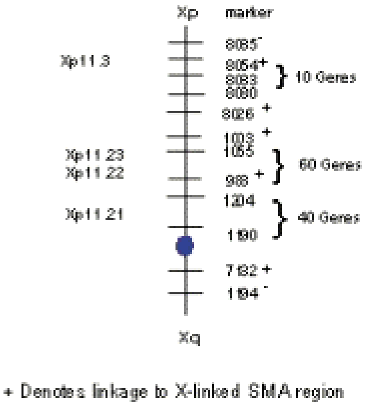

**Figure 1**

It is clear that spinal muscular atrophy is associated with neuronal apoptosis of the lower motor neurons. There is a growing number of SMA patients testing "negative" for chromosome 5q *SMN*[4] gene abnormalities. Our data suggests that XL-SMA may not be as rare as previously assumed; that families representing new mutations in the yet-to-be identified gene exist; and that patients testing negative for *SMN* mutations might instead be affected by XL-SMA. The discovery of this gene will provide major insights into these issues, and into the etiology and developmental timing of motor neuron loss.

**ACKNOWLEDGEMENTS**. This study has been generously supported by grants from the Muscular Dystrophy Association, the Patterson Trust, and Pentland-Hall Foundation of Dade County.

**REFERENCES.**

1. Online Mendelian Inheritance in Man Web page: http://www3.ncbi.nlm.nih.gov/Omim
2. Kobyashi, H., Baumbach, L., Matise, T., Schiavi, A., Greenberg, F., and Hoffman, E. (1995) Hum. Mol. Genet. 4, 1213-1216
3. Pusch, C.M., Zeitz, C., Brandau, O., et al. (2000) Nat. Genet. 26, 324-327
4. Burghes, A. (1997) Am. J. Hum Genet. 61, 9-15
